# The prognosis of bulky hepatocellular carcinoma with nonmajor branch portal vein tumor thrombosis

**DOI:** 10.1097/MD.0000000000015066

**Published:** 2019-03-15

**Authors:** Tyng-Yuan Jang, Ching-I. Huang, Ming-Lun Yeh, Zu-Yau Lin, Shinn-Cherng Chen, Wan-Long Chuang

**Affiliations:** a Hepatobiliary Division, Department of Internal Medicine, Kaohsiung Medical University Hospital, Kaohsiung Medical University, Kaohsiung; b Department of Internal Medicine, Pingtung Hospital, Ministry of Health and Welfare, Pingtung; c Faculty of Internal Medicine, School of Medicine, College of Medicine, Kaohsiung Medical University, Kaohsiung, Taiwan.

**Keywords:** nonmajor branch portal vein tumor, solitary hepatocellular carcinoma, tumor-node-metastasis

## Abstract

A bulky, solitary hepatocellular carcinoma (HCC) with nonmajor branch portal vein tumor thrombosis (PVTT) was staged as T2 in the tumor-node-metastasis (TNM) system. We aimed to evaluate the prognosis of this group of patients.

A total of 2643 patients with HCC in a medical center were consecutively enrolled. The stage of HCC was determined according to the 7th edition of American Joint Committee on Cancer staging system. Patients who were diagnosed as having solitary HCC larger than 5 cm with nonmajor portal vein thrombosis (VP1-VP2) and no lymphadenopathy or metastasis were included.Bulky HCC with nonmajor branch PVTT and without metastasis and lymphadenopathy was identified in 0.15% (4 out of 2643 patients) of the patients with HCC. Child–Pugh scores of the patients were A to B. Tumor sizes all were larger than 5 cm (mean: 6.8 ± 1.0 cm). All patients had nonmajor branch of PVTT. Three patients initially received trans-arterial chemoembolization (TACE) therapy, and 1 patient refused treatment because of old age. The response to TACE was poor: 2 patients rapidly progressed to main portal vein thrombosis, and their tumors enlarged within a half year. Only 1 patient's disease remained stable but progressed gradually 2 years later. The median survival time was 16.5 months. The 1- year, 2-year, and 3-year survival rate was 100%, 50%, and 0%, respectively.

Solitary HCC > 5 cm with PVTT of a nonmajor branch gave dismal prognoses and required aggressive treatment such as hepatic resection or combination therapy. In our opinion, it should be staged as T3 rather than a T2 in the TNM staging system.

## 1 Introduction

Hepatocellular carcinoma (HCC) causes many deaths in Taiwan and the world.^[1]^ The risk factors for HCC include chronic hepatitis B (CHB), chronic hepatitis C (CHC) infection, alcohol usage, and nonalcoholic fatty liver disease.^[2]^

There are many staging systems for HCC, including TNM staging system, the Barcelona Clinic Liver Cancer (BCLC) staging classification, Japanese Integrated Scoring (JIS), Okuda staging, and Cancer of the Liver Italian Program (CLIP) score.^[3]^

Portal vein tumor thrombosis (PVTT) was classified as VP1-VP4 in Japan, and had poor prognosis.^[4]^ An ideal staging system predicts precise prognosis and response of treatment. The TNM staging system has been used in our hospital owing to its simplicity. However, the involvement of nonmajor branch PVTT might only be staged as T2 in the TNM system, according to the 7th edition of the American Joint Committee on Cancer (AJCC).^[5]^ In another study, the larger the solitary tumor was, the poorer the prognosis.^[6]^ Therefore, we aimed to evaluate the behavior and prognosis of larger, solitary HCC (larger than 5 cm), involving nonmajor branches of PVTT (VP1-VP2) with no metastasis or lymphadenopathy.

## 2 Methods

### 2.1 Patients

From January 2010 to April 2018, a total of 2643 patients were diagnosed as having HCC at Kaohsiung Medical University Hospital in Taiwan. The diagnosis of HCC was made according to typical image (early enhancement of arterial phase and early wash out of venous phase) or pathology. The stage of HCC was according to the 7th edition of the AJCC staging system. Patients who were diagnosed as having solitary HCC larger than 5 cm with nonmajor portal vein thrombosis (VP1-VP2) and no lymphadenopathy or metastasis were included. Only 4 patients were included. The patients were regularly followed at our hospital at intervals of 1 to 3 months to evaluate the efficacy of HCC treatment. The study was conducted according to the Declaration of Helsinki. The ethical committee of the Kaohsiung Medical University Hospital approved the study.

### 2.2 Statistical analyses

Frequency was compared between groups using the Chi-squared test, with the Yate correction, or Fisher exact test. Group means, presented as the mean values standard deviation were compared using analysis of variance and Student *t* test. The procedures were performed by using the SPSS statistical package (version 20; SPSS Inc, Chicago, IL). All statistical analyses were based on 2-sided hypothesis tests with a significance level of *P* < .05.

### 2.3 Laboratory and histologic analyses

Biochemical evaluation of aspartate aminotransferase (AST) and alanine aminotransferase (ALT) levels were performed on a multichannel autoanalyzer (Hitachi Inc, Tokyo, Japan). Antibodies against hepatitis C virus (anti-HCV) were measured by 3rd-generation enzyme immunoassay (Abbott Laboratories, North Chicago, IL). Hepatitis B surface antigen (HBsAg) was measured using a standard quantitative chemiluminescent microparticle immunoassay (ARCHITECT HBsAg; Abbott Diagnostics; Finisklin Business Park, Sligo, Ireland).

## 3 Results

### 3.1 Characteristics of HCC according to AJCC stage in our hospital

From January 2010 to April 2018, a total of 2643 patients were diagnosed as having HCC in our hospital. Only 4 patients with HCC met the criteria for enrollment in the study. The rate of single HCC > 5 cm with nonmajor branch portal vein thrombosis (VP1) and no lymphadenopathy or no metastasis was only 0.15%.

### 3.2 Patient characteristics and survivals

**Figure 1:**
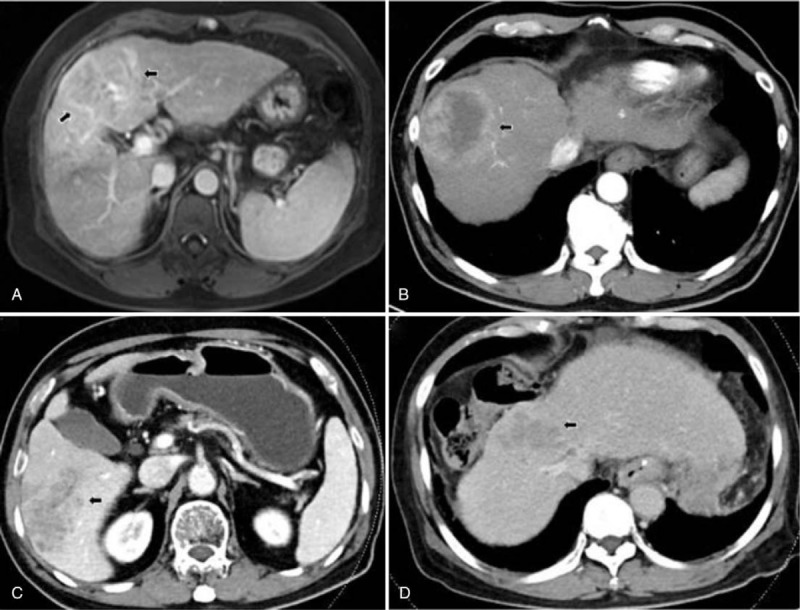
(A) 7.2-centimeter HCC at the segment 4 of liver in patient No. 1. (B) 7.5-centimeter HCC at the segment 8 of liver in patient No. 2. (C) 7.2-centimeter HCC at the segment 5–6 of liver in patient No. 3. (D) 5.3-centimeter HCC at the segment 4–8 of liver in patient No. 4.

**Figure 2:**
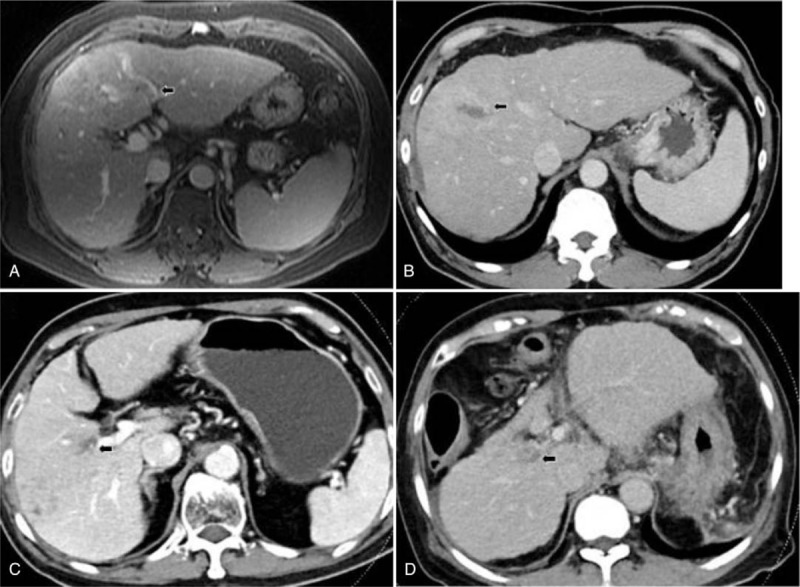
(A) Portal vein tumor thrombosis (PVTT) of left 2nd branch in patients No. 1. (B) PVTT of right 1st branch in patients No. 2. (C) PVTT of right 1st branch in patients No. 3. (D) PVTT of right 1st branch in patients No. 4.

The mean age was 68.0 years (range: 54–81 years), and males accounted for 75.0% of the sample (n = 3) (Table 1). Two patients had CHB infection, 1 patient had CHC infection, and 1 patient had alcoholism. All patients were cirrhotic. Only 1 patient received antiviral agents before diagnosis of HCC. The category of Child–Pugh score was A to B. Tumor sizes all were larger than 5 cm (mean: 6.8 ± 1.0 cm) (Table 2, Fig. 1). All patients had VP1-VP2 of PVTT (Table 2, Fig. 2). Resection of the tumor was not chosen because of poor results of indocyanine green (ICG) retention tests (patients 1 and 2) or refusal on the part of the patients (patients 3 and 4). Three patients initially received the treatment of trans-arterial chemoembolization (TACE), and 1 patient refused treatment because of old age. The response to TACE was poor: two patients (patients 1 and 2) rapidly progressed to main portal vein thrombosis and their tumors enlarged within a half year. The 2 patients subsequently received treatment with sorafenib but expired on 13th and 15th months after diagnosis of HCC. Only 1 patient (patient 4) remained with stable disease but progressed gradually 2 years later, possibly attributed to the fact that the patient refused further treatment. All patients were passed away from hepatic complications, and the median survival time was 16.5 months. The 1-, 2-, and 3-year survival rates were 100%, 50%, and 0%, respectively.

**Table 1:**
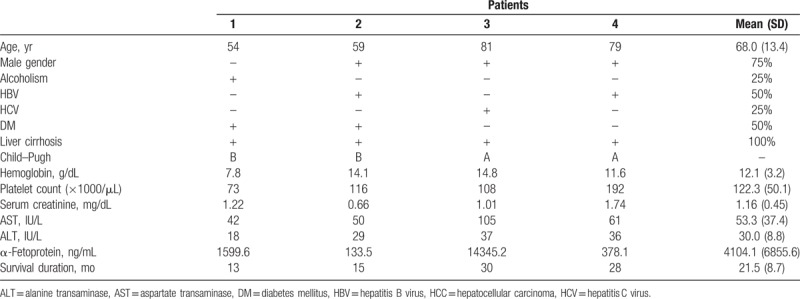
Clinical characteristics of hepatocellular carcinoma with nonmajor branch of portal vein thrombosis.

**Table 2:**
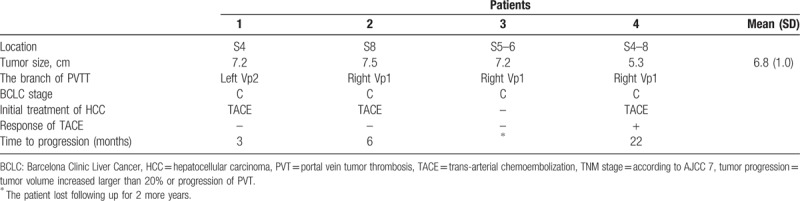
Image results of hepatocellular carcinoma with nonmajor branch of portal vein thrombosis.

The median survival of T2, T3a, and T3b stages among 2643 enrolled patients was 46.5, 15.7, and 5.9 months, respectively (Table 3). The median survival of the 4 patients was 16.5 months. The median survival was similar to T3a stage, according to the 7th edition of the AJCC, and to T3 stage according to the 8th edition of the AJCC.

**Table 3:**
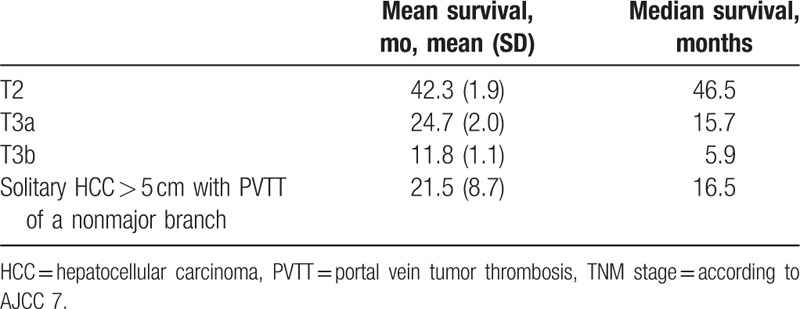
The survival of T2 and T3 stage in our hospital.

## 4 Discussion

The HCC is a common malignancy in Taiwan, and the risk factors are primarily CHC and CHB.^[1]^ The staging systems of HCC include the TNM staging system, the BCLC staging classification, JIS, Okuda staging, and CLIP score.^[3]^ The BCLC and TNM staging systems are often used in Taiwan, and the TNM system has mostly been used in patients with hepatic resection and liver transplantation.^[7–9]^ The TNM staging system lacks the evaluation of liver function, but is depicted in a more concise and delicate way.^[10]^ The involvement of PVTT might indicate poor outcome, and it could be classified to VP1-VP4 according to the Japanese staging system.^[11,12]^ Once PVTT involves a major portal vein, the prognosis becomes dismal.^[13]^ Poor prognosis was also related with a tumor size of ≥5.0 cm.^[14]^ In the present study, patients were diagnosed with solitary and large HCC with nonmajor branch PVTT, and 2 patients progressed to major branch PVTT within half a year despite treatment with TACE and sorafenib. All the 4 patients died within 3 years after diagnosis, although 1 patient remained without progression for 22 months. The rates of 3-year survival of stages II and III were 55.2% and 14.4% in our hospital, respectively. In the literature, the 5-year survival rates in the TNM staging system were 55%, 37%, and 16% in stages I, II, and III, respectively,^[15]^ similar to that of our hospital. The median survival time of stages I, II, and III was 50, 22, and 13 months, respectively.^[16]^ In the present study, the median survival time of T2, T3a, and T3b stages was 46.5, 15.7, and 5.9 months, respectively. The prognosis was dismal for these 4 patients, and it was closer to stage T3a stage in the 7th edition of AJCC or T3 stage in the 8th edition of AJCC. We suggest that bulky tumors with nonmajor branch PVTT should be staged as T3.

Solitary HCC with nonmajor branch PVTT should be considered for hepatic resection 1st if there are no contraindications.^[6]^ Our cases were not amenable to surgical treatment due to poor results of ICG retention tests. Moreover, hepatic resection still carries high rates of tumor recurrence.^[17]^ In patients with hepatectomy, independent predictors of poor survival included Child–Pugh B, cirrhosis, ICG retention rate 15 minutes >10% and microvascular invasion.^[18]^ Our cases involved macrovascular invasion, suggesting poor prognosis.

Among the patients unable to undergo surgery, TACE has some benefits in survival of patients with HCC with PVTT, although there might be partial response to tumor and PVTT.^[19,20]^ TACE combined with other treatments is an alternative choice. Targeted therapy has been introduced in recent decades, as well as effective treatments for HCC with PVTT.^[21,22]^ One systematic review showed that TACE combined with sorafenib treatment was better than TACE alone for patients with HCC with PVTT.^[23]^ Two of our cases were treated with sorafenib.

One limitation of our study was the small number of patients. However, it may reflect the rare occurrence rate of solitary HCC > 5 cm, with PVTT of nonmajor branch and without lymphadenopathy or metastasis. In the case series, its prevalence was rare (0.15%). Owning to its rarity, the survival of patients with T2 stage might not be affected. Another limitation was that the patients only received TACE and sorafenib treatment rather than TACE and radiotherapy combination therapy or surgery initially. However, the patients were elderly and had more comorbidities. In conclusion, solitary HCC > 5 cm with PVTT of nonmajor branch (VP1-VP2) are aggressive and might be staged as T3 rather than T2 in the TNM staging system. More aggressive treatment should be considered in this group of patients.

## Author contributions

**Conceptualization:** Ming-Lun Yeh.

**Data curation:** Ching-I Huang.

**Investigation:** Shinn-Cherng Chen.

**Methodology:** Shinn-Cherng Chen.

**Project administration:** Zu-Yau Lin.

**Visualization:** Wan-Long Chuang.

**Writing – Original Draft:** Jang Tyng Yuan.

**Writing – Review & Editing:** Shinn-Cherng Chen.

Jang Tyng Yuan orcid: 0000-0003-2961-130X.

## References

[1] Chen DS Hepatocellular carcinoma in Taiwan. Hepatol Res 2007;37 Suppl 2:S101–5. 17877468 10.1111/j.1872-034X.2007.00170.x

[2] Janevska D Chaloska-Ivanova V Janevski V Hepatocellular carcinoma: risk factors, diagnosis and treatment. Open Access Maced J Med Sci 2015;3:732–6. 27275318 10.3889/oamjms.2015.111 PMC4877918

[3] Faria SC Szklaruk J Kaseb AO TNM/Okuda/Barcelona/UNOS/CLIP International Multidisciplinary Classification of Hepatocellular Carcinoma: concepts, perspectives, and radiologic implications. Abdom Imaging 2014;39:1070–87. 24695938 10.1007/s00261-014-0130-0

[4] Chan SL Chong CC Chan AW Management of hepatocellular carcinoma with portal vein tumor thrombosis: Review and update at 2016. World J Gastroenterol 2016;22:7289–300. 27621575 10.3748/wjg.v22.i32.7289 PMC4997643

[5] Chun YS Pawlik TM Vauthey JN 8th Edition of the AJCC Cancer Staging Manual: pancreas and hepatobiliary cancers. Ann Surg Oncol 2018;25:845–7. 28752469 10.1245/s10434-017-6025-x

[6] Liu PH Su CW Hsu CY Solitary large hepatocellular carcinoma: staging and treatment strategy. PLoS One 2016;11:e0155588. 27176037 10.1371/journal.pone.0155588 PMC4866714

[7] Kao WY Chao Y Chang CC Prognosis of early-stage hepatocellular carcinoma: the clinical implications of substages of Barcelona Clinic Liver Cancer System Based on a Cohort of 1265 Patients. Medicine 2015;94:e1929. 26512620 10.1097/MD.0000000000001929 PMC4985433

[8] Ramacciato G Mercantini P Cautero N Prognostic evaluation of the new American Joint Committee on Cancer/International Union Against Cancer staging system for hepatocellular carcinoma: analysis of 112 cirrhotic patients resected for hepatocellular carcinoma. Ann Surg Oncol 2005;12:289–97. 15827681 10.1245/ASO.2005.03.098

[9] Vauthey JN Ribero D Abdalla EK Outcomes of liver transplantation in 490 patients with hepatocellular carcinoma: validation of a uniform staging after surgical treatment. J Am Coll Surg 2007;204:1016–27. 17481532 10.1016/j.jamcollsurg.2006.12.043

[10] Sherman M Hepatocellular carcinoma: screening and staging. Clin Liver Dis 2011;15:323–34. 21689616 10.1016/j.cld.2011.03.003

[11] Chan SL Mo FK Johnson PJ Prospective validation of the Chinese University Prognostic Index and comparison with other staging systems for hepatocellular carcinoma in an Asian population. J Gastroenterol Hepatol 2011;26:340–7. 21261725 10.1111/j.1440-1746.2010.06329.x

[12] Levi Sandri GB Ettorre GM Colasanti M Hepatocellular carcinoma with macrovascular invasion treated with yttrium-90 radioembolization prior to transplantation. Hepatobiliary Surg Nutr 2017;6:44–8. 28261594 10.21037/hbsn.2017.01.08 PMC5332219

[13] Katagiri S Yamamoto M Multidisciplinary treatments for hepatocellular carcinoma with major portal vein tumor thrombus. Surg Today 2014;44:219–26. 23591833 10.1007/s00595-013-0585-6 PMC3898334

[14] Chan AC Fan ST Poon RT Evaluation of the seventh edition of the American Joint Committee on Cancer tumour-node-metastasis (TNM) staging system for patients undergoing curative resection of hepatocellular carcinoma: implications for the development of a refined staging system. HPB (Oxford) 2013;15:439–48. 23659567 10.1111/j.1477-2574.2012.00617.x PMC3664048

[15] Vauthey JN Lauwers GY Esnaola NF Simplified staging for hepatocellular carcinoma. J Clin Oncol 2002;20:1527–36. 11896101 10.1200/JCO.2002.20.6.1527

[16] Liu C Duan LG Lu WS Prognosis evaluation in patients with hepatocellular carcinoma after hepatectomy: comparison of BCLC, TNM and Hangzhou criteria staging systems. PLoS One 2014;9:e103228. 25133493 10.1371/journal.pone.0103228 PMC4136742

[17] Zhong JH Rodriguez AC Ke Y Hepatic resection as a safe and effective treatment for hepatocellular carcinoma involving a single large tumor, multiple tumors, or macrovascular invasion. Medicine 2015;94:e396. 25621684 10.1097/MD.0000000000000396 PMC4602643

[18] Lim C Mise Y Sakamoto Y Above 5 cm, size does not matter anymore in patients with hepatocellular carcinoma. World J Surg 2014;38:2910–8. 25099682 10.1007/s00268-014-2704-y

[19] Xue TC Xie XY Zhang L Transarterial chemoembolization for hepatocellular carcinoma with portal vein tumor thrombus: a meta-analysis. BMC Gastroenterol 2013;13:60. 23566041 10.1186/1471-230X-13-60 PMC3626696

[20] Tawada A Chiba T Ooka Y Efficacy of transarterial chemoembolization targeting portal vein tumor thrombus in patients with hepatocellular carcinoma. Anticancer Res 2014;34:4231–7. 25075052

[21] Kokudo N Hasegawa K Akahane M Evidence-based Clinical Practice Guidelines for Hepatocellular Carcinoma: The Japan Society of Hepatology 2013 update (3rd JSH-HCC Guidelines). Hepatol Res 2015;45:123–7. 10.1111/hepr.12464 25625806

[22] Kudo M Kitano M Sakurai T General rules for the clinical and pathological study of primary liver cancer, nationwide follow-up survey and clinical practice guidelines: the outstanding achievements of the Liver Cancer Study Group of Japan. Dig Dis 2015;33:765–70. 26488173 10.1159/000439101

[23] Zhang X Wang K Wang M Transarterial chemoembolization (TACE) combined with sorafenib versus TACE for hepatocellular carcinoma with portal vein tumor thrombus: a systematic review and meta-analysis. Oncotarget 2017;8:29416–27. 28177886 10.18632/oncotarget.15075 PMC5438741

